# Atypical/Nor98 Scrapie Infectivity in Sheep Peripheral Tissues

**DOI:** 10.1371/journal.ppat.1001285

**Published:** 2011-02-10

**Authors:** Olivier Andréoletti, Leonor Orge, Sylvie L. Benestad, Vincent Beringue, Claire Litaise, Stéphanie Simon, Annick Le Dur, Hubert Laude, Hugh Simmons, Séverine Lugan, Fabien Corbière, Pierrette Costes, Nathalie Morel, François Schelcher, Caroline Lacroux

**Affiliations:** 1 UMR INRA ENVT 1225, Interactions Hôtes Agents Pathogènes, Ecole Nationale Vétérinaire de Toulouse, Toulouse, France; 2 Laboratório Nacional de Investigação Veterinária, Lisboa, Portugal; 3 National Veterinary Institute, Oslo, Norway; 4 INRA UR892, Virologie et Immunologie Moléculaires, INRA, F-78350 Jouy-en-Josas, France; 5 CEA, Service de Pharmacologie et d'Immunoanalyse, IBiTec-S, DSV, CEA/Saclay, Gif sur Yvette cedex, France; 6 VLA Weybridge, ASU, Addlestone, Surrey, United Kingdom; University of Alberta, Canada

## Abstract

Atypical/Nor98 scrapie was first identified in 1998 in Norway. It is now considered as a worldwide disease of small ruminants and currently represents a significant part of the detected transmissible spongiform encephalopathies (TSE) cases in Europe. Atypical/Nor98 scrapie cases were reported in ARR/ARR sheep, which are highly resistant to BSE and other small ruminants TSE agents. The biology and pathogenesis of the Atypical/Nor98 scrapie agent in its natural host is still poorly understood. However, based on the absence of detectable abnormal PrP in peripheral tissues of affected individuals, human and animal exposure risk to this specific TSE agent has been considered low. In this study we demonstrate that infectivity can accumulate, even if no abnormal PrP is detectable, in lymphoid tissues, nerves, and muscles from natural and/or experimental Atypical/Nor98 scrapie cases. Evidence is provided that, in comparison to other TSE agents, samples containing Atypical/Nor98 scrapie infectivity could remain PrP^Sc^ negative. This feature will impact detection of Atypical/Nor98 scrapie cases in the field, and highlights the need to review current evaluations of the disease prevalence and potential transmissibility. Finally, an estimate is made of the infectivity loads accumulating in peripheral tissues in both Atypical/Nor98 and classical scrapie cases that currently enter the food chain. The results obtained indicate that dietary exposure risk to small ruminants TSE agents may be higher than commonly believed.

## Author Summary

Following the bovine spongiform encephalopathy (BSE) crisis and the identification of its zoonotic properties, a sanitary policy has been implemented based on both eradication of transmissible spongiform encephalopathies (TSE) in food-producing animals and exclusion of known infectious materials from the food chain. Atypical/Nor98 scrapie is a prion disease of small ruminants identified worldwide. Currently it represents a significant part of the TSE cases detected in Europe. The restricted tissue distribution of Atypical/Nor98 scrapie agent in its natural host and the low detected prevalence of secondary cases in affected flocks meant that it is believed to be a poorly transmissible disease. This has led to the view that Atypical/Nor98 scrapie is a spontaneous disorder for which human and animal exposure risk remains low. In this study we demonstrate that in affected individuals, Atypical/Nor98 scrapie agent can disseminate in lymphoid tissues, nerves, and muscles, challenging the idea that it is a brain-restricted infectious agent. Evidence for the deficiencies in the current methods applied for monitoring Atypical/Nor98 scrapie is provided that would indicate an underestimation in the prevalence in the general population and in the affected flocks. These elements challenge the hypothesis on the biology of this recently identified TSE agent.

## Introduction

Transmissible spongiform encephalopathies (TSE), or prion diseases, are fatal neurodegenerative disorders occurring in sheep (scrapie), cattle (bovine spongiform encephalopathy - BSE), or humans (Creutzfeldt-Jakob disease - CJD). The key event in TSE is the conversion of a normal cellular protein (PrP^c^) into an abnormal isoform (PrP^Sc^) which accumulates in tissues from infected individuals [1]. PrP^Sc^ is currently considered to be the only TSE biochemical marker. According to the prion concept, abnormal PrP would be the causative agent of TSE [2].

Following the BSE crisis and the identification of its zoonotic properties [3], [4], the control of human and animal exposure to TSE agents has become a priority. A sanitary policy has been implemented based on both eradication of TSE in food producing animals and exclusion of known infectious materials from the food chain.

In 1998 an Atypical/Nor98 Scrapie was identified in Norwegian sheep; the PrP^Sc^ signature was partially PK resistant and displayed a multi-band pattern as showed by Western Blot (WB) that contrasted with those normally observed in small ruminants TSE cases [5]. After 2001 and the implementation of active TSE surveillance plans, a number of similar cases were identified in most EU members states as well in other countries, like Canada, USA and New Zealand [6]. The transmissibility of Atypical/Nor98 agent has been demonstrated in both rodent models (transgenic animals expressing the ovine *Prnp* gene) [7] and sheep [8], [9].

Currently Atypical/Nor98 Scrapie represents a significant part of the TSE cases identified in the EU small ruminant population, where its prevalence was estimated to range between 5 to 8 positive small ruminants per 10,000 tested per year [10].

Atypical/Nor98 scrapie cases have different biological features from those observed in other small ruminants TSE [5], [6]. Sheep susceptibility to TSE is strongly controlled by polymorphisms on the gene (*Prnp*) encoding for PrP protein [11], [12]. The homozygous and heterozygous ARR sheep are considered to be strongly resistant to both the classical scrapie [11], [12] and the cattle BSE agents [13]. This resistance has been the basis of a large scale genetic selection policy aiming at the control of TSE diseases by increasing the frequency of the ARR allele in general population and restocking affected flocks with ARR animals. In Atypical/Nor98 scrapie the sheep genetic susceptibility is significantly different from what is observed in classical TSE forms, with homozygous and heterozygous ARR allele carriers being susceptible to the disease [14], [15], [16], [17], [18].

Information about the tissue distribution of Atypical/Nor98 scrapie agent in the host species is limited [5], [19], [20], [21] but research findings indicate that no detectable abnormal PrP has been found in peripheral tissues and that the infectious agent could be restricted to the central nervous system. This key feature led to consider that dietary exposure risk to Atypical/Nor98 scrapie is low. The apparent limited spreading of Atypical/Nor98 scrapie in the organism of affected individuals is also an argument supporting the hypothesis that this agent has restricted abilities to spread into the environment or between individuals. PrP^Sc^ detection generally correlates with the presence of infectivity [1], [22] but infectivity has been reported in the absence of detectable PK resistant PrP [23]. In this study, we investigate the potential presence of TSE infectivity in peripheral tissues (lymphoid organs, striated muscles and nerves) of Atypical/Nor98 scrapie from naturally and experimentally infected sheep. We then compared the relative infectivity level present in peripheral tissues with those estimated in similar tissues from classical scrapie affected sheep.

## Results

Atypical/Nor98 scrapie field cases (n = 7) collected in three different countries (Portugal, Norway and France) were investigated for the presence of PrP^Sc^ and infectivity in lymphoid tissues and central nervous system (Table 1). These sheep were of various *Prnp* genotypes including those associated with high susceptibility to Atypical/Nor98 scrapie (homozygous or heterozygous A_136_F_141_R_154_Q_171_– AHQ) or resistance (homozygous and heterozygous ARR) to classical scrapie or BSE [11], [13]. Amongst the seven cases, five were identified by the active surveillance program either at rendering plant or slaughter house and two through the passive surveillance network (clinical suspects). Three of these cases were identified in fallen stock animals collected in three independent flocks where an Atypical/Nor98 scrapie case had previously been identified (Portugal).

**Table 1:** Abnormal PrP (PrP^Sc^) and infectivity in central nervous system and lympho-reticular system of natural Atypical/Nor98 scrapie incubating or affected animals bearing various genotypes at codons 136, 141, 154 and 171 of the *Prnp* gene.

						PrP^Sc^			tg*338* transmission		
Case	Genotype	Origin	Age	Category	Tissues	WB	ELISA	IHC	Positive mice	Incubation period in days (mean +/−SD)*	Estimated infectious titre(IC ID_50_ in tg*338*/*g*)
1	AFRQ/AFRQ	FR	6 years	clinical	Cerebral cortex	pos	pos	pos	6/6	209+/−12	10^8.7^
					Brainstem	neg	neg	pos^++^	6/6	256+/−14	10^5.5^
					Prescapular LN	neg	neg	neg	3/5	334+/−10	ND
2	ALRQ/ARR	PT	6 years	fallen stock (additional case)	Cortex	pos	pos	pos	6/6	231+/−17	10^6.7^
					Retropharyngeal LN^+^	neg	neg	ND	5/6	349+/−76	ND
3	ARR/ARR	PT	8 years	fallen stock (additional case)	Cerebral cortex	pos	pos	pos	6/6	221+/−19	10^6.7^
					Retropharyngeal LN^+^	neg	neg	ND	4/6	348+−37	ND
4	AFRQ/VRQ	PT	9 years	fallen stock (additional case)	Cerebral cortex^+^	pos	pos	pos	6/6	239+/−28	10^6^
					Retropharyngeal LN^+^	neg	neg	ND	0/6	>650	
5	AHQ/AHQ	NO	3,5 years	clinical	Cerebellum	pos	pos	pos	5/5	224+/−21	10^6.7^
					Parotideal LN	neg	neg	neg	2/2	285–298*	10^4.4^
6	AHQ/AHQ	NO	7,5 years	slaughter	Cerebellum	pos	pos	pos	5/5	248+/−15	10^5.8^
					Popliteal LN	neg	neg	neg	1/10	401*	ND
7	ARR/ARR	NO	7 years	slaughter	Cerebellum	pos	pos	pos	5/5	279+/−46	10^5.8^
					Thoracic spinal cord	neg	neg	neg	5/5	346+/−85	10^2.9^
					Prescapular LN	neg	neg	neg	0/6	>650	

Two different methods were applied to detect PrP^Sc^ presence in tissue homogenate that were used for mice inoculation: WB (TeSeE WB kit – BioRad, using SHa31 as anti-PrP antibody) and ELISA (TeSeE Sheep and Goat– BioRad). Additionally when formalin fixed tissue was available, PrP^Sc^ immunohistochemistry was also carried out (using 8G8 antibody). (^++^) indicate a case were minimal PrP^Sc^ labelling was observed after the exam of a serial series of slides. In some cases (^+^), the tissue homogenates were heated (60°C −10 min) in order to destroy contaminating bacteria before bioassay. Such heat treatment might have reduced infectivity level in these samples in an unknown proportion. Mice were considered positive when abnormal PrP deposition was detected in brain. Incubation periods are presented as mean +/−SD (in days) except for that dilution with which less than 50% of mice were found positive. In that case (^*^) incubation periods of the positive mice are individually presented. In the different tissues, infectious titres were estimated on the basis of incubation period in mice (Figure 4). This estimation was only performed when the attack rate was 100% and data from more than 5 animals were available.

ND: not done.

In each of these natural Atypical/Nor98 cases, PrP^Sc^ accumulation could be detected in different brain areas by WB and/or Immunohistochemistry (IHC). Conversely, no abnormal PrP deposits were evidenced in any of the investigated lymphoid organs (Table 1).

**Figure 1:**
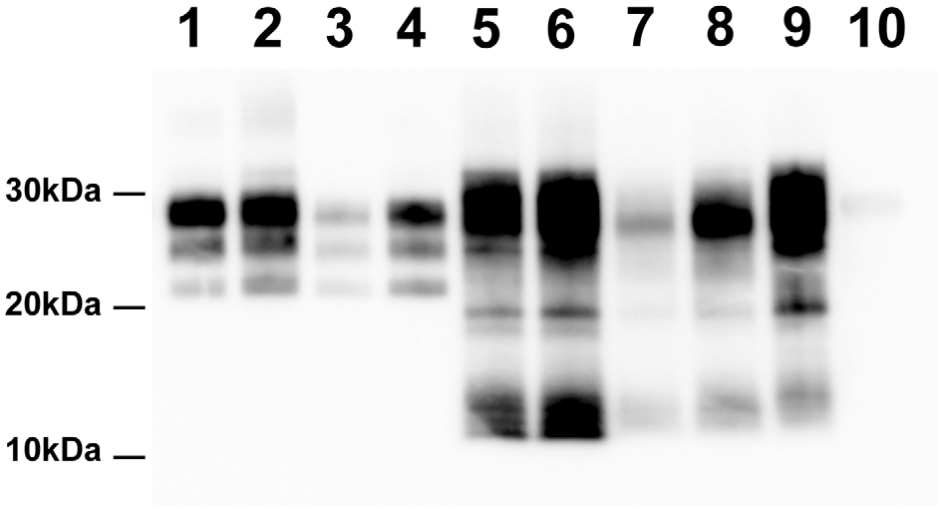
PrP^Sc^ Western Blot detection in sheep and *tg338* mice inoculated with Atypical/Nor98 scrapie and classical scrapie tissues (10% tissue homogenate). **Lane 1**: posterior brainstem from a Langlade classical scrapie affected sheep (case 10). **Lane 2**: brain from a *tg338* mouse inoculated with striated muscle (semi-membranous) from the same sheep (case 10). **Lane 3**: posterior brainstem from a PG127 classical scrapie orally inoculated sheep (case 12). **Lane 4**: brain from a *tg338* mouse inoculated with striated muscle (extra-ocular motor muscle) from the same PG127 classical scrapie affected sheep (case 12). **Lane 5**: cerebral cortex from an AFRQ/AFRQ Atypical/Nor98 scrapie natural case (case 1). **Lane 6**: AHQ/AHQ sheep (cerebellum-case 9) intra-cerebrally challenged with natural atypical scrapie (case 1). **Lane 7 to 9**: brain homogenates from *tg338* mice inoculated with peripheral tissues from atypical scrapie cases. **Lane 7**: retropharyngeal lymph node (case 2). **Lane 8**: sciatic nerve (case 9). **Lane 9**: striated muscle (case 8). No PrP^Sc^ was observed in control *tg338* mice inoculated with retropharyngeal LN from a negative control sheep (**Lane 10**).

**Figure 2:**
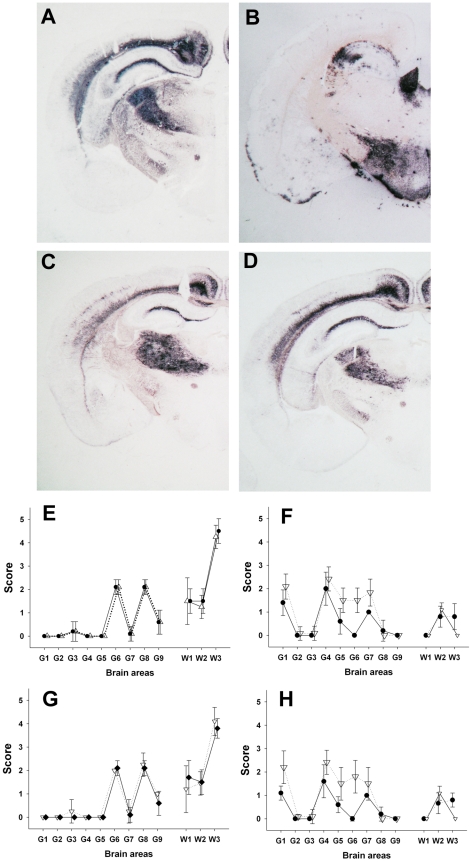
PrP^Sc^ distribution pattern and lesion profile in *tg338* mice inoculated with Atypical/Nor98 scrapie and classical scrapie sheep tissues. (**A–D**) PET Blot (SHa31 antibody – NBT/BCIP black deposits- bar: 200 µm) of brain coronal section (thalamus level) from *tg338* inoculated with (**A**) AHQ/AHQ atypical scrapie cerebellum (case 9), (**B**) posterior brainstem from VRQ/VRQ classical scrapie (PG127- case 12), (**C**) retropharyngeal lymph node from a ARR/ARR natural atypical case at preclinical stage of the disease (case 3), (**D**) striated muscle from a AFRQ/ARQ experimental atypical case (case 8) at clinical stage of the disease. (**E–H**) Lesion profile (vacuolar changes) in *tg338* mice inoculated with: (**E**) Natural (case 3 - △) and experimental (case 9- ●) cerebral cortex from atypical scrapie cases, (**F**) Langlade (case 10-▽) and PG127 (case 12- ●) VRQ/VRQ posterior brainstem homogenates, (**G**) brachial nerve from an AFRQ/ARQ experimental atypical case (case 8-◆) and AFRQ/AFRQ natural atypical case (case 1-▽), (**H**) striated muscle from Langlade (case 10- ▽- semi-membranous) and PG127 (case 12- ●-psoas) classical scrapie affected sheep.

Bioassay of brain homogenates prepared from these seven sheep into transgenic mice that over-express the VRQ allele of ovine PrP (*tg338*) [7] were positive for TSE. Surprisingly, despite the absence of detectable PrP^Sc^, the lymphoid tissue homogenates from five out of the seven cases were positive for TSE in *tg338* mice. The attack rate in mice challenged with lymphoid tissues was lower and the clinical onset was delayed compared to mice inoculated using CNS homogenate (Table 1). Brains collected in clinically affected mice inoculated either with lymphoid tissues or brain homogenates displayed a similar PrP^Sc^ WB pattern (Figure 1: lanes 5, 6, 7 – Figure S2), PrP^Sc^ deposits distribution and vacuolar lesion profile (Figure 2A, 2C, 2E- – Figure S2). All these features were identical to those previously reported in *tg338* mice inoculated with a panel of Norwegian, French [7] and UK [24] Atypical/Nor98 scrapie isolates. These phenotypic features were clearly different from those associated to two distinct classical scrapie isolates (Figure 1: lanes 1–4 and Figure 2B, 2F, 2H). Infectivity was demonstrated in lymphoid tissues from one out of the two investigated ARR/ARR Atypical/Nor98 scrapie cases.

Samples collected in two experimental Atypical/Nor98 scrapie cases were also analyzed; these were from an AHQ/AHQ (case 9) and an AFRQ/ARQ (case 8) sheep which had been intra-cerebrally challenged with an AFRQ/AFRQ field Atypical/Nor98 scrapie isolate (case 1, Figure 1: lane 5). These sheep had an incubation period of 964 and 2240 days respectively. In both animals' CNS, PrP^Sc^ displayed a WB banding pattern that was characteristic [14] of Atypical/Nor98 scrapie (Figure 1: lane 6 – Figure S2). In none of the investigated peripheral tissues PrP^Sc^ could be detected (Table 2). In the *tg338* bioassay, infectivity was shown in some but not all tested lymphoid tissues, and in striated muscle and peripheral nerves from both cases (Table 2 – Figure 1: lanes 8–9 – Figure S2). Incubation periods were prolonged in comparison to brain homogenates and the attack rate was lower than 100%. The phenotypes of the propagated prions in *tg338* mice (WB banding pattern, vacuolar lesion profile and PET Blot PrP^Sc^ distribution in brain) were identical to the one observed with natural Atypical/Nor98 scrapie isolates (Figure 1: lanes 5, 7, 9- Figure 2C, 2E, 2G).

**Table 2:** PrP^Sc^ and infectivity in tissues from experimental (IC challenged) Atypical/Nor98 scrapie cases and natural or experimental (oral or intra-cerebral challenge) classical scrapie cases.

					PrP^SC^			Bioassay results		
TSE isolate	Case	Case origin	Genotype	Tissue	ELISA	WB	IHC	Positive mice	Incubation period in days (mean +/−SD)	Estimated infectious titre (ID_50_ IC in tg*338*/g)
Atypical/Nor98 scrapie	8	Experimental intracerebral	AFRQ/ARQ	Cerebellum	pos	pos	pos	6/6	204+/−5	10^8.7^
				Ileal LN	neg	neg	neg	0/6	-	
				Prescapular LN	neg	neg	neg	1/5	445*	ND
				Brachial nerve	neg	neg	neg	3/5	369+/−20	ND
				External ocular muscle	neg	neg	neg	5/6	409+/−66	ND
	9	Experimental intracerebral	AHQ/AHQ	Cerebral cortex	pos	pos	pos	6/6	219+/−4	10^8.3^
				Ileal LN	neg	neg	neg	0/6	-	
				Sciatic nerve	neg	neg	neg	3/6	516+/−133	ND
				External ocular muscle	neg	neg	neg	2/6	370–450*	ND
Langlade Classical scrapie	10	Natural	VRQ/VRQ	Posterior brain stem	pos	pos	pos	6/6	221+/−20	10^6.8^
				Spleen	pos	ND	pos	6/6	431+/−32	10^5.5^
				Ileal LN	pos	ND	pos	6/6	436+/−66	10^5.5^
				External ocular muscle	pos	neg	pos	6/6	427+/−29	10^5.6^
				Semi membranous muscle	pos	neg	pos	6/6	481+/−46	10^5.4^
	11	Experimental intracerebral	VRQ/VRQ	Posterior brain stem	pos	pos	pos	6/6	229+/−14	10^6.8^
				Psoas muscle	pos	neg	pos	6/6	389+/−63	10^5.8^
PG 127 Classical scrapie	12	Experimental oral	VRQ/VRQ	Posterior brain stem	pos	pos	pos	6/6	64+/−4	10^6.6^
				Ileal LN	pos	ND	pos	6/6	78+/−3	10^5.5^
				External ocular muscle	pos	ND	pos	6/6	81+/−2	10^5.1^
				Psoas muscle	pos	ND	pos	6/6	87+/−7	10^4.5^
	13	Experimental intracerebral	VRQ/VRQ	Posterior brain stem	pos	pos	pos	6/6	61+/−1	≥10^6.6^
				Tonsil	pos	ND	pos	6/6	79+/−1	10^5.4^
				External ocular muscle	pos	ND	pos	6/6	77+/−2	10^5.5^

Animals were sampled at clinical stage of the disease. Mice were considered positive when abnormal PrP deposition was detected in brain. Incubation periods are presented as mean +/−SD except for that dilution with which less than 50% of mice were found positive. In that case (*) incubation periods of the positive mice are individually presented. ND: not done.

Two different methods were applied to detect PrP^Sc^ presence in tissue homogenate that were used for mice inoculation: WB (TeSeE WB kit – BioRad, using SHa31 as anti-PrP antibody) and ELISA (TeSeE Sheep and Goat– BioRad). Additionally when formalin fixed tissue was available PrP^Sc^ immunohistochemistry was also carried out (using 8G8 antibody). Infectious titres in the different tissues were estimated on the basis of incubation period in mice (Figure 4). This calculation was only performed when the attack rate was 100% and data from more than 5 animals were available.

**Figure 3:**
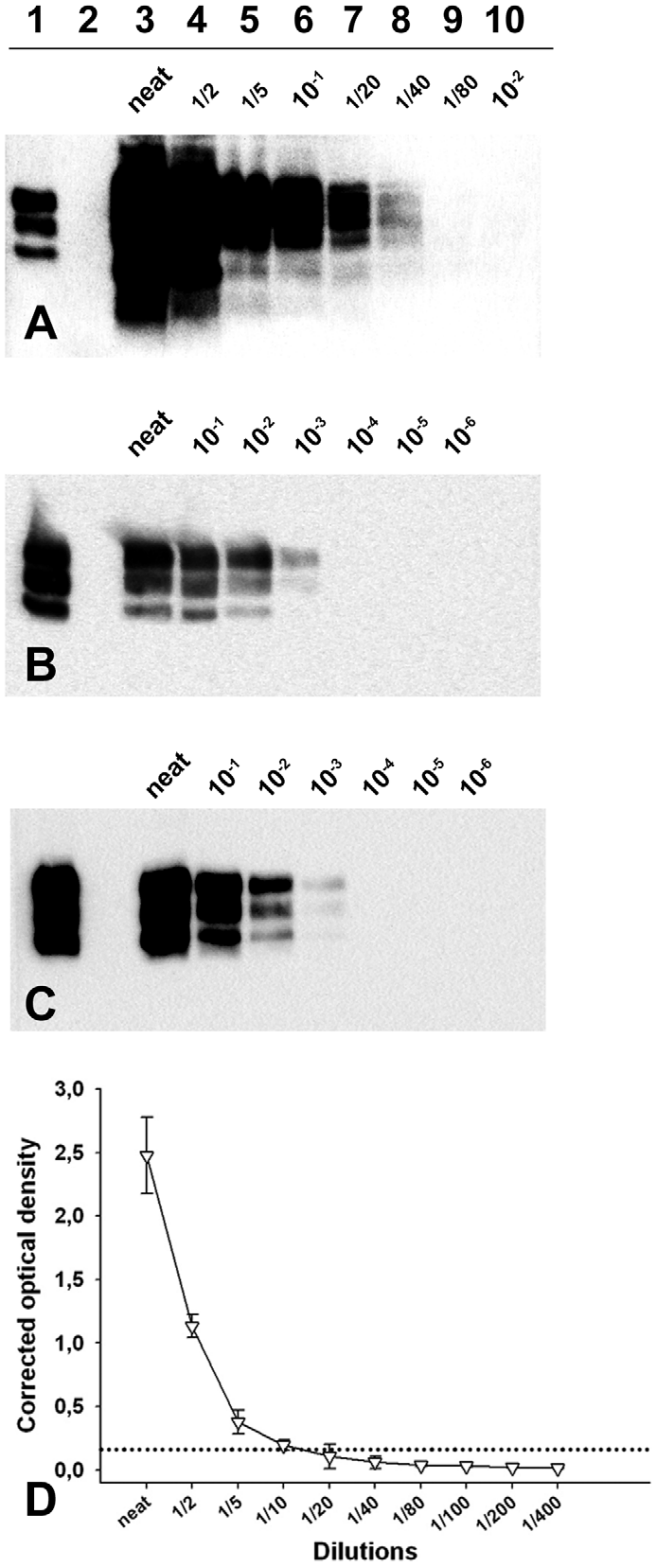
PrP^Sc^ detection limit of a Langlade classical scrapie isolate, a PG127 classical scrapie isolate and an Atypical/Nor98 scrapie isolate. Dilution series from (**A, D**) Atypical/Nor98 scrapie isolate (case 9- cerebral cortex), (**B**) Langlade–case 10- posterior brainstem) and (**C**) PG127 (case 12- posterior brainstem) classical scrapie isolate, that were prepared in negative sheep brain homogenate. The tissue homogenates (see methods) are the same than the one used for endpoint titration in *tg338* mice (Table 3). The samples were processed for PrP^Sc^ Western-blotting (**A–C**, TeSeE WB kit – BioRad, anti-PrP SHa31 antibody). After extraction (25 mg of brain equivalent material), the re-suspended pellet was either entirely loaded on lanes (**A:** all lanes – **B** and **C**: Lanes 4–6) or diluted in Laemmli's buffer before loading (**B**: lane 3, 1/50 – Lane 4, 1/10 – **C**: lane 3, 1/20). These dilutions were necessary to avoid a saturation of the signal. A classical scrapie control was included on the three gels to calibrate the signal (**Lane 1**). (**D**) The atypical/Nor98 scrapie isolate dilution serie (case 9: cerebral cortex) was tested using a commercially available rapid TSE test (TeSeE Sheep and Goat - BioRad) used for field TSE screening in small ruminants. Three different aliquots of each dilution were independently tested. The extractions were carried out. Results are presented as the mean +/− SD corrected optical density values. The cut off value (0.162 OD– dotted lines) was established as the mean of four negative control optical densities + 0.150 OD.

A similar experiment was performed using peripheral and CNS tissues from natural or experimental classical scrapie cases at clinical stage of the disease. This study involved two distinct classical scrapie agents (Langlade and PG127), which can be distinguished on the basis of their lesion profile in *tg338* mice (Figure 2F). The inoculation of peripheral tissues homogenates from animals infected with those classical scrapie agents into *tg338* resulted in a 100% attack rate transmission, but with prolonged incubation period by comparison to mice inoculated with CNS samples (Table 2). For both isolates, PrP^Sc^ WB banding pattern (Figure 1: lanes 1–4 – Figure S2), vacuolar lesion (Figure 3F, 3H – Figure S2) observed in mice inoculated with CNS and peripheral tissues were identical. As previously described and conversely to Atypical/Nor98 scrapie, PrP^Sc^ could be detected in the investigated lymphoid organs, and striated muscle [25], [26], [27], [28] from all the four classical scrapie affected animals involved in this study (Table 2).

In order to determine the cause of our incapacity to detect abnormal PrP in the peripheral tissues of Atypical/Nor98 scrapie cases that contain infectivity, classical scrapie (cases 10 and 12) and Atypical/Nor98 scrapie (cases 1, 8, 9) brain homogenates dilution series were prepared and processed for a OIE registered PrP^Sc^ detection WB (TeSeE WB Kit – BIORAD), a PrP^Sc^ ELISA detection assay (TeSeE Sheep and Goat^ -^ BIORAD) (Figure 3 and Figure S1) and bioassays in t*g338* mice (Table 3).

**Table 3:** Classical scrapie and Atypical/Nor98 scrapie isolates endpoint titration and infectious titre in ovine PrP transgenic mice (*tg338*).

	Langlade classical scrapie (Case 10)		PG127classical scrapie (Case 12)		ARQ/ARQ Atypical/Nor98 scrapie (case 14)		ARR/ARR Atypical/Nor98 scrapie (case 15)		AFRQ/AFRQ Atypical/Nor98 scrapie (Case 1)		AFRQ/ARQ Atypical/Nor98 scrapie (Case 8)		AHQ/AHQ Atypical/Nor98 scrapie (Case 9)	
Dilution	Positive mice	Incubation period	Positive mice	Incubation period	Positive mice	Incubation period	Positive mice	Incubation period	Positive mice	Incubation period	Positive mice	Incubation period	Positive mice	Incubation period
neat	6/6	221+/−20	6/6	64+/−4	7/7	224+/−10	10/10	184+/−4	6/6	209+/−12	6/6	204+/−5	6/6	219+/−4
10^−1^	6/6	348+/−16	6/6	76+/−3	ND		ND		ND		ND		ND	
10^−2^	12/12	481+/−32	6/6	87+/−2	ND		ND		ND		ND		ND	
10^−3^	10/12	594+/−34	6/6	97+/−5	ND		ND		ND		ND		ND	
10^−4^	7/12	713+/−43	3/6	110+/−4	6/6	258+/−18	6/6	272+/−23	ND		ND		ND	
10^−5^	3/12	805, 824, 852*	2/6	117–121*	6/6	294+/−41	6/6	300+/−17	6/6	308+/−34	6/6	321+/−28	6/6	315+/−51
10^−6^	0/12	>900	0/6	>300	6/6	329+/−34	5/6	311+/−43	5/6	353+/−75	3/6	334+/−77	6/6	345+/−55
10^−7^			0/6	>300	2/6	360–412*	1/6	392*	1/6	451*	0/6	>550	2/6	368–532*
10^−8^					1/6	392*	0/6	>720	0/6	>650	0/6	>550	0/6>	>620
Infectious titre (ID_50_ IC t*g338*/g)	10 ^6.8^		10^6.6^		10^9.5^		10^9.1^		10^9.1^		10^8.7^		10^9.5^	

Incubation periods are expressed in days (mean +/−SD) except in cases where less than 3 animals were positive (*) for which individual incubation periods are presented. Each mouse received 20 µl intracerebrally. The Langlade scrapie isolate homogenate (12.5% weight/volume) was prepared with posterior brainstem from case 10 (Figure 3 and Table 2). These data were already used in a previous publication [32]. The PG127 isolate homogenate (10% weight/volume) was prepared using posterior brainstem from case 13 (Figure 3 and Table 2). ND: not done.

**Figure 4:**
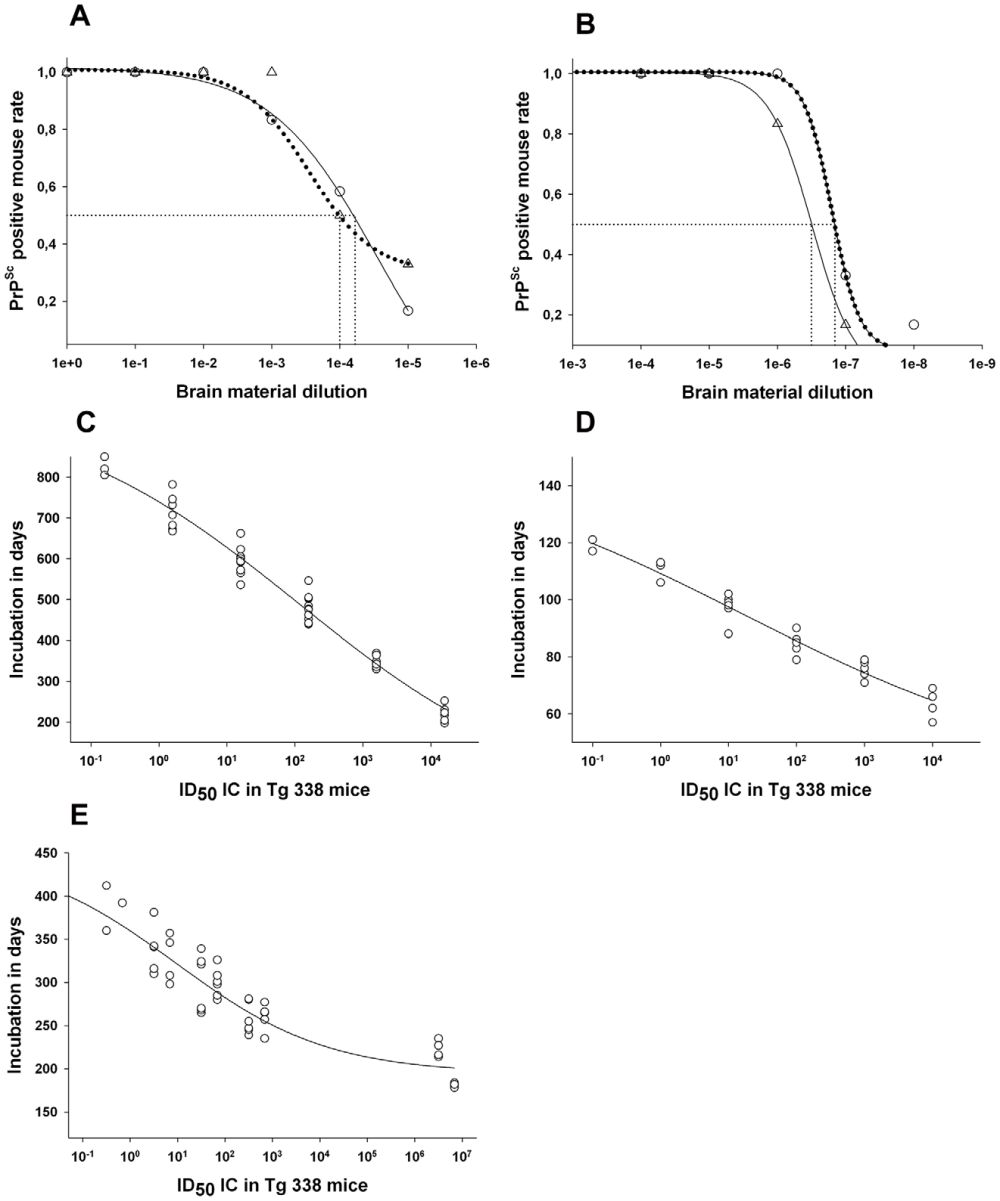
Infectivity titre in reference brain sample from classical and Atypical/Nor98 affected ewes. (**A–B**) Intra-cerebral endpoint titration in a *tg338* mouse model of CNS homogenate (20 µl per mice), (**A**) prepared from terminally scrapie affected sheep with Langlade isolate (12.5% homogenate - case 10 - ○) and with PG127 isolate (10% homogenate- case 13- △) – (**B**) an ARQ/ARQ (10% homogenate - case 14- ○) and an ARR/ARR (10% homogenate -case 15- △) atypical scrapie cases identified in field through active epidemiosurveillance program. Such titration allowed the determination of the infectious dose 50 (ID_50_) of the reference brain samples (Table 3). (**D–E**) Variation of the incubation period as a function of the infectious dose inoculated intra-cerebrally in *tg338* mice. (**C**): Langlade isolate, (**D**): PG127 isolate and (**E**): atypical scrapie isolate. To establish these diagrams, the individual incubation periods and the number of ID_50_ inoculated in each mouse were plotted. The number of ID_50_ is obtained by multiplying the titre of the inoculum (ID50 IC in *tg338* per gram of tissue) per the amount of tissue inoculated in each mice. On this basis, a four parameter logistic regression was computed (Sigma Plot) to provide a curve associating the incubation period and the number of ID_50_ inoculated with the observed incubation period.

For the 5 Atypical/Nor98 cases a 10% weight/volume homogenate was used. Case 1: cerebral cortex. Case 8: cerebellum. Case 9: cerebral cortex. Case 14 cerebellum. Case 15 cerebellum (Figure 3, supplemental Figure S2 and Table 1). Cases 14 and 15 are Atypical/Nor98 field cases independent from those presented in Tables 1 and 2 and were titrated (10% weight/volume homogenate) in different premises than the other isolates. Infectious titre (number of ID_50_ IC in t*g338* per gram of tissues) were determined from this endpoint titration using the Spearman-Karber's approach (for example see Figure 4A, 4B).

According to the endpoint titration, the infectious titre in the Langlade and PG127 classical scrapie isolates were estimated to be respectively 10^6.8^ ID_50_ IC *tg338* per gram and 10^6.6^ ID_50_ IC t*g338* per gram (Table 3- Figure 4A). All the titrated Atypical/Nor98 scrapie cases (n = 5, see Table 3) that we investigated displayed substantially higher infectious titres, ranging between 10^8.7^ and 10^9.5^ ID_50_ IC *tg338*/g (Table 3-Figure 4 B).

For the two classical scrapie isolates, WB detected PrP^Sc^ to a dilution of 10^3^ (positive detection on 25 µg of brain equivalent material) which corresponded to 10^2.2^ (Langlade isolate) and 10^2^ (PG127 isolate) ID_50_ IC *tg338* (Figure 3). For the AHQ/AHQ Atypical/Nor98 scrapie isolate (case 9), PrP^Sc^ WB detection limit was the 1/80 dilution (312 µg of brain starting material) (Figure 3A) which corresponded to 10^6^ IC ID_50_ in *tg338* (Figure 4E). Similar results were obtained with the two other atypical scrapie isolates (cases 1 and 8) for which the detection limits of PrP^Sc^ assays were respectively equivalent to 10^5.6^ and 10^5.5^ ID_50_ IC *tg338* (Table 3 and Figure S1). These results indicate that PrP^Sc^ detection assays currently used for field TSE testing could have a dramatically lower intrinsic sensitivity for identifying Atypical/Nor98 scrapie agent than classical scrapie agent.

As previously described [29], [30], [31], [32], [33], [34], the end point infectivity titration data that was generated with the two different types of classical scrapie agents (case 10 and case 12) and the Atypical/Nor98 scrapie cases (cases 14 and 15) were used to fit the best logistic regression models correlating the incubation periods in *tg338* mice with the infectious dose (Figure 4). These models were then applied to estimate the infectious content in the different tissues using the incubation periods in t*g338* mice (Tables 1 and 2).

By this approach, the infectious titre of three atypical scrapie samples (cases 1: cerebral cortex - case 8: cerebellum – case 9: cerebral cortex) were estimated respectively 10^8.7^, 10^8.7^, and 10^8.3^ ID_50_ IC *tg338*/gram (Tables 1 and 2). The measured infectious titre in the same three samples, by endpoint titration in *tg338*, were respectively 10^9.1^, 10^8.7^ and 10 ^9.5^ ID_50_ IC *tg338*/gram (Table 3).

In the investigated Atypical/Nor98 scrapie cases, the infectious load in muscle and lymphoid tissues samples from sheep affected were close to the sensitivity of our bioassay (10^2,7^ ID_50_ IC per gram of tissue); ie about 10^6^ fold lower than the infectivity level measured (by endpoint titration) or estimated (on the basis of the incubation periods) in the same amount of brain prepared from clinically affected sheep (Tables 1 and 2).

In sheep affected by the two different classical scrapie agents the incubation period recorded in *tg338* inoculated with lymphoid tissues and striated muscle were consistent with infectious titre about 10 fold lower than the one measured in brains from those terminally affected animals (Table 2).

## Discussion

Bioassay endpoint titration is considered as the most accurate method for determining the TSE infectivity titre in tissues. Although regarded as less accurate, dose-response relationships have been used as a method for infectivity estimation when endpoint titration data are not available; in such an approach, the incubation period observed in the inoculated mice is used to estimate the infectious titre of the samples tested [29], [30], [31], [32], [33], [34]. In this study, the dose-response approach was used to estimate the infectious titre in various peripheral tissues from Atypical scrapie/Nor98 and classical scrapie affected sheep. For mice inoculated with peripheral tissue homogenates, the standard curve established using reference CNS homogenates was used, but it was established that the lesion profile was identical in the mice inoculated with the peripheral tissue and the CNS. Although it could be hypothesized that the nature of the peripheral tissue inoculated would impact on the observed incubation length in mice (matrix effect) and consequently on the estimated infectious titre, Dickinson et al. [29], [30] demonstrated that in conventional mice the dose-response obtained with the spleen and the brain from ME7 infected mice are similar.

Together these elements indicate, that even if the peripheral tissues infectious titre reported are ‘estimates’, they provide a good guide to the relative infectivity levels that are present in the Atypical scrapie/Nor98 and classical scrapie cases' brain and peripheral tissues.

The presence of PrP^Sc^ and infectivity in small ruminant's peripheral tissues affected with natural classical scrapie or experimental BSE is well established [25], [27], [35], [36], [37]. It is generally considered that peripheral tissues like lymphoid tissues and striated muscle contain much lower levels of prion than CNS from terminally affected animals. This concept is the basis of the statutory measures aiming at limiting the entry of small ruminants TSE agents into the food chain. Tissues considered to be the most infectious (named Specific Risk Material) are systematically discarded from consumption, but tissues that would potentially contain only a low level of infectivity might enter the food chain due to the feasibility/practicality of removing them.

In this study, the estimated infectivity level in skeletal muscle and lymphoid tissues from animals (n = 4) affected with two different classical scrapie isolates did reach up to 1/10 (weight/weight) of the infectivity found in the CNS from terminally affected sheep. These values are higher than those expected from previous work. This could be explained by the fact that previously available data on prion quantities in peripheral tissues of small ruminants (in particular those related to striated muscle) relied on biochemical measurement of PrP^Sc^ amount [26] and the cell types accumulating PrP^Sc^ and the composition of these tissues may have impact on the PrP^Sc^ recovery yield. Also, if in some classical scrapie cases a 3–4 log10 infectivity difference was reported between CNS and some lymphoid tissues using bioassay in conventional mice, in other classical scrapie cases, the same study reported that infectivity in lymphoid tissue was only 1 to 10 fold lower than in CNS [27].

The classical scrapie cases that were investigated in this work cannot be assumed to be representative of all field diversity as only four animal cases of highly susceptible genotypes were used. However, the results indicate that exposure risk to such TSE agents through the unrestricted entry in the food chain of potentially infectious tissues would be significantly higher than previously thought.

In most countries, the identification of Atypical/Nor98 scrapie was a consequence of the implementation of an active surveillance for TSE consisting in random testing for PrP^Sc^ presence in brainstem of a fraction of fallen or healthy culled small ruminants [10]. In Atypical/Nor98 scrapie cases, the sensitivity of PrP^Sc^ detection tests that are used for initial field screening or confirmation of TSE cases is debated. Several authors reported failure to detect PrP^Sc^ in some CNS areas like the obex area [5], [6], [20] from known affected animals or discrepancies in results when applying different diagnostic tests to a same sample [6], [10].

The results obtained in this study by comparing the analytical sensitivity of biochemical PrP^Sc^ detection (using an OIE registered WB method and a validated rapid screening test for TSE detection, in small ruminants) and bioassay indicated that CNS samples that would contain up to 10^7.4.^–10^7.7^ ID50/g of Atypical/Nor98 scrapie (according to *tg338* IC bioassay) could remain negative for PrP^Sc^ detection. In field, Atypical/Nor98 scrapie cases (Table 1) PrP^Sc^ positive WB was observed in CNS samples in which infectious titre was estimated (on the basis of incubation period) to be higher than 10^5.8^ ID50/g IC in *tg338*. Such discrepancies might reflect an individual variability of the PrP^Sc^ WB detection limits between atypical scrapie cases. It might alternatively be the consequence of a relative imprecision in estimating the titre of low infectious doses by the incubation period bioassay method.

In contrast to Atypical/Nor98 scrapie cases, using two different classical agents the WB PrP^Sc^ detection sensitivity limit was about 10^2^ ID_50_ IC in *tg338* (ie a tissue with a titre of 10^3.7^ ID_50_/g IC in *tg338*). These differences strongly support the contention that diagnostic assays based on PrP^Sc^ detection have lower performance for identifying Atypical/Nor98 scrapie cases than classical scrapie cases. It is consequently highly probable that a significant number of Atypical/Nor98 cases remain undetected by field testing, leading to an underestimation of Atypical/Nor98 scrapie prevalence in the small ruminant population. It is however not possible on the sole basis of this study to evaluate the importance of such underestimation.

The under detection of Atypical/Nor98 scrapie in the field due to the sensitivity of the current PrP^Sc^ based approach would also impact on understanding of the biology of this TSE agent.

While under natural conditions, classical scrapie is known to transmit between individuals, the analysis of data collected through the active TSE surveillance program seemed to indicate that Atypical/Nor98 scrapie could be poorly or not transmissible at all. This is based on the lack of statistical difference of the observed Atypical/Nor98 frequencies between the general population and the flocks where a positive case had been identified [38], [39]. The lower ability to detect Atypical Scrapie incubating animals using the PrP^Sc^ based methodologies means that this conclusion should be considered with caution.

Atypical/Nor98 cases are identified in older animals in comparison to classical scrapie [6], [40]. The lack of PrP^Sc^ detection in peripheral tissues of reported cases suggested that Atypical/Nor98 scrapie agent could be restricted to CNS. This is supportive of the hypothesis that Atypical/Nor98 scrapie could be a spontaneous disorder of PrP folding and metabolism occurring in aged animals without external cause [6], [38].

However, this hypothesis is questioned by the evidence reported here that a negative PrP^Sc^ testing result could be observed in animals harbouring high infectious titre in their brain and that the infectious agent can be present in peripheral tissues of Atypical/Nor98 scrapie incubating sheep. TSE are considered to be transmitted following oral exposure; initial uptake is followed by a peripheral replication phase which is generally associated with a dissemination of the agent in the lymphoid system and the deposition of large amounts of PrP^Sc^. This peripheral replication phase is later followed by the entry of the infectious agent into the CNS through the autonomic nervous system [25], [27], [35], [36]. However, in several situations, like BSE in cattle [41], [42], [43] or classical scrapie in ARR heterozygote sheep [44], [45], the involvement of secondary lymphoid system is marginal, which does not preclude central neuro-invasion through the autonomic nervous system [46]. It could be proposed that Atypical Scrapie/Nor98 might occur following oral exposure to a TSE agent, which would spread marginally in lymphoid tissues before neuro-invasion. The slow propagation of Atypical Scrapie/Nor98 in its host (long incubation period) and the impaired detection sensitivity level of PrP^Sc^ based assays would explain the apparent old age of detected cases.

The results presented here are insufficient to rule out the hypothesis of a spontaneous/non contagious disorder or to consider this alternative scenario as a plausible hypothesis. Indeed, the presence of Atypical scrapie/Nor98 infectivity in peripheral tissues could be alternatively due to the centripetal spreading of the agent from the CNS. However, our findings point out that further clarifications on Atypical/Nor98 scrapie agent biology are needed before accepting that this TSE is a spontaneous and non contagious disorder of small ruminants. Assessing Atypical/Nor98 scrapie transmissibility through oral route in natural host and presence in placenta and in colostrum/milk (which are considered as major sources for TSE transmission between small ruminants) [28], [32] will provide crucial data.

The presence of infectivity in peripheral tissues that enter the food chain clearly indicates that the risk of dietary exposure to Atypical/Nor98 scrapie cannot be disregarded. However, according to our observations, in comparison to the brain, the infectious titres in the peripheral tissues were five log10 lower in Atypical/Nor98 scrapie than in classical scrapie. Therefore, the reduction of the relative exposure risk following SRM removal (CNS, head, spleen and ileum) is probably significantly higher in Atypical/Nor98 scrapie cases than in classical scrapie cases. However, considering the currently estimated prevalence of Atypical/Nor98 scrapie in healthy slaughtered EU population [10], it is probable that atypical scrapie infectivity enters in the food chain despite the prevention measures in force.

Finally, the capacity of Atypical/Nor98 scrapie agent (and more generally of small ruminants TSE agents) to cross species barrier that naturally limits the transmission risk is insufficiently documented. Recently, the transmission of an Atypical/Nor98 scrapie isolate was reported into transgenic mice over-expressing the porcine PrP [47]. Such results cannot directly be extrapolated to natural exposure conditions and natural hosts. However, they underline the urgent need for further investigations on the potential capacity of Atypical/Nor98 scrapie to propagate in other species than small ruminants.

## Methods

### Ethics statement

All animal experiments were performed in compliance with our institutional and national guidelines, in accordance with the European Community Council Directive 86/609/EEC. The experimental protocols were approved by the INRA Toulouse/ENVT and by the Norwegian ethics committees.

### Atypical/Nor98 and classical scrapie natural cases

The natural classical scrapie case (case 10) included in this experiment was a Romanov sheep born and bred in the Langlade flock where a natural scrapie epidemic has been occurring at a high incidence since 1993 [11].

Natural atypical scrapie cases were identified though active or passive surveillance programs in France, Norway and Portugal (Table 1). The Portuguese cases were identified in three independent flocks where an atypical case had already been identified in the past (additional cases). In all cases, PrP genotype was obtained by sequencing the Exon 3 of the *Prnp* gene as previously described [14]. In each case, the polymorphisms at codons 136 (A/V), 154 (H/R) and 171 (R/Q), which have been demonstrated to strongly influence the susceptibility to TSE in sheep are indicated [48]. Additionally the presence of a phenylalanine at codon 141 (F/L), which has been shown to impact on the susceptibility to atypical/Nor98 scrapie, was indicated [14], [49].

### Atypical/Nor98 scrapie and classical scrapie experimental cases

Two sheep (one 12 months old AFRQ/ARQ (case 8) and one 14 months old AHQ/AHQ (case 9)) selected in a field flock were IC challenged with French AFRQ/AFRQ Atypical Scrapie (case 1) (Table 2). The animals were euthanized when showing clear clinical signs at respectively 2224 and 964 days post inoculation.

TSE-free Poll-Dorset sheep (VLA- Weybridge- UK) were used for intracerebral inoculation with Langlade isolate (case 10, inoculum derived from a VRQ/VRQ natural isolate), or PG127 isolate (inoculum derived from a VRQ/VRQ experimental case). Animals were killed when displaying evident clinical signs at respectively 380 days and 160 days post inoculation. Oral challenge was performed in 6–10 months old TSE-free New Zealand cheviot sheep. Animals were dosed with 5 g equivalent of brain material (1% brain homogenate in glucose) derived from an experimentally VRQ/VRQ affected sheep (PG127 isolate). Animals were culled at clinical stage of the disease (200 days post inoculation).

### Tissue samples collection, homogenate preparation

All tissues were collected using disposable equipment (forceps and scalpels). The different field and experimental cases were sampled on different dates and/or places. Different instrument sets and containers were used for collecting, transporting and storing each sample. Finally, in all cases, peripheral tissues were collected before CNS to further reduce the risk of cross contamination. In natural Atypical/Nor98 cases, the nature of the tissues collected under TSE sterile conditions might have varied according to the country and date of collection. In all cases, CNS and at least one lymph node were available. In both Atypical/Nor98 and classical scrapie experimental cases, a large panel of tissues (including Central Nervous System, Peripheral Nervous System, digestive tract wall, muscle) was collected under TSE sterile conditions.

From the available samples, tissues homogenates (20% stock material) were prepared in Norway (Norwegian cases) or in France (French natural and experimental cases and Portuguese cases). The list of processed samples is given in Tables 1 and 2. In each case disposable equipment was used to manipulate the tissues. 20% tissues homogenates were prepared using single use grinding microtubes (Precess 48 - BioRad) and filtered through a 25 gauge needle (single use syringe). The tissue homogenates were then aliquoted (in 2 ml and 5 ml tubes) and stored at −80°C. Peripheral tissues homogenates and CNS homogenates were prepared separately.

### Tissue processing and immunohistochemistry (IHC) detection

This method was performed as previously described [50]. PrP^Sc^ IHC detection was first performed using 8G8 antibody raised against human recombinant PrP protein and specifically recognising the 95–108 amino acid sequence (SQWNKP) of the PrP protein.

For each sample a negative serum control was included, in which the primary antibody was either omitted or replaced by purified mouse IgG2a serum.

### PrP^Sc^ Western-blot detection (WB)

An OIE registered Western blot kit (TeSeE Western Blot, BioRad) was used following the manufacturer's recommendations. For each sample, 250 µl of 10% brain homogenate were submitted to PrP^Sc^ extraction. The obtained pellet was denaturated in Laemmli's buffer (15* µ*l) before being loaded neat or diluted (Figure 4) on a 12% acrylamide gel, and submitted to electrophoresis and blotting. Immunodetection was performed using SHa31 which recognizes the 145–152 sequence of PrP (YEDRYYRE). Peroxidase activity was revealed using ECL substrate (Pierce) [26].

### ELISA PrP^Sc^ detection

A commercially available TSE detection test (TeSeE Sheep and Goat - BioRad) was used according to manufacturer's recommendations. In summary, five hundred µL of the 20% homogenate were incubated for 10 min at 37°C with 500 µL of buffer A containing proteinase K. PrP^sc^ was recovered as a pellet after addition of 500 µL of buffer B and centrifugation for 5 min at 20 000 g at room temperature. Supernatant was discarded and tubes dried. Finally, the pellet was denatured in buffer C (5 min at 100°C) and 1:6 diluted in R6 reagent before distribution into the wells [28], [51].

### Paraffin-embedded tissue blot (PET blot)

PET blots were performed using a method previously described [52], [53]. Immunodetection was carried out using SHa31 monoclonal antibody (4 µg/mL), followed by application of an alkaline phosphatase labeled secondary antibody (Dako reference D0314 – 1/500 diluted). Enzymatic activity was revealed using NBT/BCIP substrate chromogen.

### Bioassay

Bioassay experiments were carried out in ovine VRQ PrP transgenic mice (*tg338*), which are considered to be highly efficient for the detection of sheep scrapie infectivity [54]. At least six mice were intra-cerebrally inoculated with each sample (20 µL).

Prior to inoculation, homogenates were diluted (final concentration 10% or 12.5%) in 5% glucose sterile solution. Each homogenate was then tested for bacteria presence (blood gelose overnight 37°C culture) and non sterile homogenates were submitted to a heat treatment (60°C – 10 min). Heat treated samples are identified in Table 1. The impact of such heat treatment on atypical scrapie infectivity is currently unknown.

Portuguese and French cases' inoculations were carried out in UMR INRA ENVT 1225 (Toulouse, France) facilities while Norwegian cases were inoculated at the NVI (Oslo, Norway). Peripheral tissues and CNS homogenates were inoculated on different days in order to avoid any risk of cross contamination. In some cases, tissues autolysis resulted in the death of some animals inoculated which explain the low number of mice for some isolates. Mice were monitored daily until the occurrence clinical signs of TSE. Mice were culled when they started to show locomotor disorders and any impairment in their capacity to feed. CNS samples were individually collected. A part of the brain (cerebral cortex) was frozen for PrP^Sc^ Western blot testing (TeSeE WB kit- BioRad) and the other part of the brain was formalin fixed for vacuolar brain lesion profiling [55] and PrP^Sc^ PET-Blotting.

### Reference Central Nervous System samples end point titration

Five different isolates were endpoint titrated in t*g338* mice, by inoculating intra-cerebrally (20 µl) successive 1/10 dilutions of CNS homogenate in groups of *tg338* mice (6 or 12 mice). The material used for the titration was 10% brain homogenate except for the Langlade isolate (12.5% homogenate).

Two classical scrapie inocula used for sheep inoculation (Langlade: case 10, posterior brain stem - PG127: case 12, posterior brain stem) were titrated in UMR INRA ENVT 1225. The titration of the Langlade material was already published in a previous study [32].

Two confirmed atypical scrapie isolates (one from an ARQ/ARQ Norwegian sheep: case 14, cerebellum and one from a French ARR/ARR sheep: case 15, cerebellum) were titrated in INRA Jouy-en-Josas. These isolates correspond to two atypical cases originally described in the Le Dur *et al.* study in which they were respectively identified as Lindos and DS8 [7].

Three additional atypical scrapie isolates (AFRQ/AFRQ: case 1, cerebral cortex- AHQ/AHQ: case 9, cerebral cortex– AFRQ/ARQ: case 8, cerebellum) were titrated in UMR INRA ENVT 1225.

The infectious titre (Infectious Dose 50) of the brain homogenates was determined by the Spearman-Kärber's method [56].

### Estimation of infectious titre on the basis of incubation period

For each isolate, the incubation periods recorded in individual *tg338* and the number of ID_50_ inoculated to each mice (number of ID_50_ per 20 µL of the inoculated homogenate) (derived from Table 3) were plotted on a graph. On the basis of this data a four parameter logistic regression function was computed (Sigmaplot). This function was then used to estimate the infectious titre (number of Infectious Dose 50) contained in tissue samples on the basis of the incubation period observed in t*g338* mice [29], [30], [31], [32], [33], [34].

### PrP^Sc^ versus bioassay analytical sensitivity

CNS homogenate dilutions series from three different Atypical/Nor98 scrapie cases (case 1: cerebral cortex – case 8: cerebellum – case 9: cerebral cortex) were prepared by successive dilutions in negative brain homogenate.

The prepared dilutions were: 1/2, 1/5, 1/10, 1/20, 1/40, 1/80, 1/100, 1/200, 1/400, 1/800, 10^−3^, 10^−4^, 10^−5^, 10^−6^, 10^−7^, 10^−8^. The dilutions series were tested for PrP^Sc^ using TeSeE Sheep and Goat ELISA test and the WB as previously described in the text.

The neat sample and 10^−5^ to 10^−7^ dilutions from the same series were inoculated in groups of 6 t*g338* in order to assess the infectious titre (see paragraph: Reference Central Nervous System samples endpoint titration).

Dilutions series of CNS homogenates were prepared from the Langlade scrapie (case 10: posterior brainstem) and PG127 (case 12: posterior brainstem) homogenates that were endpoint titrated in *tg338* mice (Table 3). For these dilutions an aliquot of the 20% stock homogenate (stored at −80°C) was used as starting material. The dilution series (neat, 10^−1^, 10^−2^, 10^−3^, 10^−4^, 10^−5^, 10^−6^, 10^−7^) were then tested by WB (TeSeE WB kit – BioRad).

## Supporting Information

Figure S1 **PrP^Sc^ detection limit in Atypical/Nor98 scrapie isolates.** Dilution series from two atypical Atypical/Nor98 scrapie isolates (case 1: cerebral cortex, and case 8: cerebellum) were prepared in negative sheep brain homogenate. The tissue homogenates (see methods) are the same than those used for endpoint titration in *tg338* mice (Table 3). (**A–B**) The samples were processed for PrP^Sc^ using an OIE registered Western-blotting technique (TeSeE WB kit – BioRad, anti-PrP Sha31 antibody). After extraction (25 mg of brain equivalent material) the re-suspended pellet was entirely loaded on gels. (**A**) case 1: cerebral cortex. (**B**) case 8: cerebellum. The same classical scrapie control than in Figure 3A-3C was included on the two gels to calibrate the signal (**Lane 1**). (**C**) The same dilution series were tested using a commercially available rapid TSE test (TeSeE Sheep and Goat - BioRad) used for field TSE screening in small ruminants. Three different aliquots of each dilution were independently tested. The extractions were carried out. Results are presented as the mean +/− SD corrected optical density values. The cut off value (0.162 OD– dotted line) was established as the mean of four negative control optical densities +0.150 OD. (○: case 1. △: Case 8).
(0.43 MB TIF)

Figure S2 **PrP^Sc^ Western Blot profile and vacuolar lesion profile in t** *g338* **mice inoculated with various tissues from an Atypical/Nor98 scrapie and classical scrapie affected sheep.** (**A**) PrP^Sc^ Western Blot profile. **Lane 1**: posterior brainstem from a PG127 classical scrapie affected sheep (case 12). **Lane 2**: brain from a 700 days old negative control *tg338* mouse. **Lane 3**: cerebral cortex from an AFRQ/AFRQ Atypical scrapie/Nor98 natural case (case 1). **Lanes 4–7**, brains from *tg338* mice inoculated with various tissues homogenates prepared from an AFRQ/ARQ Atypical scrapie/Nor98 experimental case (case 8). **Lane 4**: cerebellum. **Lane 5**: prescapular lymph node. **Lane 6**: brachial nerve. **Lane 7**: external ocular motor muscle. **Lane 8**: brain from a *tg338* mouse inoculated from a PG127 classical scrapie affected sheep (case 12). (**B**) PrP^Sc^ Western Blot profile. **Lane 1**: posterior brainstem from a PG127 classical scrapie isolate sheep (case 12). **Lane 2**: brain from a 700 days old negative control *tg*3*38* mouse. **Lanes 3–9**: brains from *tg338* mice inoculated with various tissues homogenates prepared from two VRQ/VRQ PG127 classical scrapie cases (case 12 and 13). **Lane 3**: case 12, posterior brainstem. **Lane 4**: case 12, ileal lymph node. **Lane 5**: case 12, external ocular muscle. **Lane 6**: case 12, psoas muscle. **Lane 7**: case 13, posterior brainstem. **Lane 8**: case 13, tonsil. **Lane 9**: case 13, external ocular motor muscle. **Lane 10**: posterior brainstem from a PG127 classical scrapie sheep (case 12). (**C–D**) Lesion profile (vacuolar changes) in *tg338* mice inoculated with homogenates prepared from different tissues (**C**) an experimental Atypical/Nor98 AFRQ/ARQ scrapie case (case 8) or (**D**) an experimental VRQ/VRQ PG127 classical scrapie experimental case (case 12). (**C**) ○ cerebellum ◆: brachial nerve, ▽: external ocular motor muscle. (**D**) ●: posterior brainstem, ▽: ileal lymph node, ○: external ocular motor muscle.
(0.53 MB TIF)
